# The Prospective Effect of Green Tea versus Pomegranate Peels Extracts on Submandibular Salivary Glands of Albino Rats after Methotrexate Administration (Histological and Immunohistochemical Study)

**DOI:** 10.1155/2024/3290187

**Published:** 2024-01-03

**Authors:** Rasha M. Taha, Ghada A. Abdel-Latif, Rania H. Said

**Affiliations:** ^1^Oral Biology Department, Faculty of Dentistry, Suez Canal University, Ismailia, Egypt; ^2^Oral and Maxillofacial Pathology Department, Faculty of Dentistry, Suez Canal University, Ismailia, Egypt; ^3^Department of Oral and Maxillofacial Diagnostic Sciences, College of Dentistry and Hospital, Taibah University, Medina, Saudi Arabia; ^4^Oral Pathology Department, College of Dental Medicine, Umm Alqura University, Makkah, Saudi Arabia

## Abstract

**Background:**

There is curiosity in the use of substances that can stop cell damage. Antioxidants are substances that can prevent free radicals from damaging cells, and they can be used to treat and avoid a wide variety of illnesses.

**Objectives:**

The current investigation set out to evaluate the histological changes brought on by a single high dose of methotrexate in the submandibular glands of rats treated with green tea and pomegranate peel extract, both are well-known as antioxidants.

**Materials and Methods:**

Forty-eight healthy Albino rats were used in the current study. Animals were divided into six groups. Group 1: Vehicle group which is control group, Group 2: methotrexate treated group, Group 3: green tea control group, Group 4: pomegranate peel extract control group, Group 5: green tea + methotrexate group, and Group 6: pomegranate peel extract + methotrexate group. Rats of all groups were left 1 week after the end of the treatment. Cervical dislocation was used to kill all of the rats. Samples were gained from the rats' submandibular salivary glands of different groups for histological and immunohistochemical evaluation.

**Results:**

Green tea + methotrexate group showed improvement in the histological picture of the submandibular salivary gland compared to methotrexate group and the pomegranate peel extract + methotrexate group.

**Conclusion:**

The antioxidant activity of green tea is more potent than that of pomegranate peels extract in blocking methotrexate that induced cytotoxicity in the submandibular salivary glands of rats. As a result, it can be administered to people undergoing cancer treatment as a safeguard for their salivary glands.

## 1. Introduction

Chemotherapy for cancer has cytotoxic effects that are not selective and can harm healthy tissues as well as cancer cells. The degree of harm varies according to treatment modality, dose, and duration [1]. The antimetabolite medication methotrexate (MTX) is available. Although it is commonly used to treat a wide variety of cancers and nonmalignant conditions like ectopic pregnancies, rheumatoid arthritis, psoriasis [2], Crohn's disease, and ulcerative colitis [3], its effectiveness is limited due to its side effects [4]. Also, it is used as an immunosuppressive drug to prevent organ rejection [5]. MTX is the standard treatment because it is effective, inexpensive, and widely used and has anti-inflammatory and antiproliferative actions that are widely used in clinics as an adjuvant for several types of cancer therapies [6].

The main way MTX makes cells toxic is by making the antioxidant enzyme defense system less effective. This makes cells vulnerable to reactive oxygen species (ROS). Therefore, different antioxidants may prevent cell damage caused by oxidative stress [7].

It has been shown that natural products like green tea (GT) can protect against the harmful effects of oxidative stress. *Camellia sinensis*, the plant that produces GT is high in polyphenolic flavonoids, which are powerful antioxidants. GT polyphenol epigallocatechin gallate (EGCG) has been shown to have powerful antioxidant, antiapoptotic, anti-inflammatory, and auto antigen-inhibitory effects. Because of its potential to prevent diseases brought on by oxidative stress [8] and to restore healthy salivary gland cell proliferation, it has been the subject of extensive research [9].

The pomegranate, or *Punica granatum L*. (Punicaceae), is a fruit that is native to the Middle East. Extracts of pomegranate's peels, flowers, and seeds have been touted for their purported biological efficacy in treating a variety of ailments, including infections, oxidative stress, diabetes, and cancer [10]. Histopathological changes and some oxidative stress noticed induced in the kidney and liver of female albino rats were reportedly prevented by a methanolic extract of pomegranate peel, according to a few studies. These outcomes might originate from pomegranate peel extract's antioxidant and antiapoptotic properties [11].

MTX has been used for a long time, but because it has side effects, researchers are always looking for ways to make it less harmful. In MTX treatment, there is a dose–response relationship, but the best dose is different for each person. There are not many sources that explain what a high dose of MTX does to the submandibular salivary gland, which is one of the body's most important organs. Apoptosis thought to be one of the main causes of the sudden damage to the submandibular salivary gland treated by MTX in cancer [12].

A key part of keeping the structure and function of the submandibular gland normal is the complicated relationship between cell growth, differentiation, and death. The survival of tissue is an important part of healing from damage caused by chemotherapy [13, 14]. Ibrahim et al. [15] noticed that it took a drug less time to approach its optimum concentration in submandibular saliva secretion than in parotids. Because of this, the goal of the current research was to compare the anticytotoxic effects of GT extract 40 mg/kg daily and pomegranate peels extract (PPE) (500 mg per kg) on the submandibular glands cells of Albino rats with high dose of MTX through histological and immunohistochemical evaluated methods to demonstrate cell growth and death and figure out how much damage or protection it offered.

## 2. Materials and Methods

### 2.1. Sample Size Determination

The sample size was determined with the help of G ^*∗*^Power version 3.1.9.2, Faul et al.'s [16] University of Kiel, Germany. Copyright (c.) 1992 [16].

**Figure 1:**
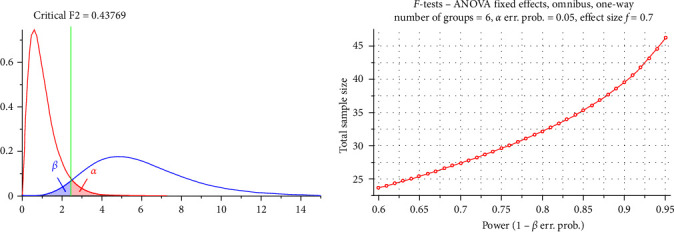
A chart showing *F*-tests and ANOVA test effects.

Estimated sample size (*n*) should be 48 samples, and they were split evenly among six groups; effect size (*f*) was 0.70 at the 0.05*α* and 0.05*β* levels (power = 95%), (eight samples each) (Figure 1).

### 2.2. Animals and Study Design

Forty-eight Albino rats with good health and weighing 250 ± 25 mg were bought from the animal house, Faculty of Dentistry, Suez Canal University, and utilized in the study; they were housed in an environment temperature (21–24°C), exposed to a 12 hr light and dark cycles, and provided with easy accessibility to water and food under laboratory conditions. The animals were divided as following.

Group 1 (vehicle control group) (eight rats): Two doses of distilled water were given orally by intragastric gavage needle twice daily for 5 days as part of the vehicle control group, and physiological saline (0.9% NaCl) was injected intraperitoneally in place of MTX 72 hr following the start of the experiment.

Group 2 (MTX group) (eight rats): After 72 hr of receiving the distilled water orally by intragastric gavage needle twice daily for 5 days, the animals were given an intraperitoneal injection of MTX (80 mg/kg) [12].

Group 3 (GT extract treated control group) (eight rats): GT extract (20 mg/kg) was given orally twice per day (total 40 mg/kg/day) [17] which introduced orally by intragastric gavage needle for 5 days, and MTX was replaced with physiological saline (0.9% NaCl) intraperitoneally after 72 hr [12].

Group 4 (PPE treated control group) (eight rats): PPE (250 mg/kg) was given orally via intragastric gavage needle twice daily (total 500 mg/kg) [18]. For 5 days, and after 72 hr, MTX was replaced with physiological saline (0.9% NaCl) intraperitoneally

Group 5 (GT extract + MTX) (eight rats): Extract of GT (20 mg/kg) [17] was introduced by oral intragastric gavage needle twice daily during the course of the 5-day testing period, while MTX (80 mg/kg) was given via intraperitoneal injection after 72 hr [12].

Group 6 (PPE + MTX group) (eight rats): The PPE (250 mg/kg) [18] was given orally twice daily, whereas the MTX (intraperitoneal injection) was given after 72 hr [12].

The following materials were used:

1. MTX vials purchased from Mylane SAS Corporation, France.
2. *GT extract* was prepared at the Faculty of Science, Suez Canal University.
3. PPE was prepared at the Faculty of Science, Suez Canal University.

### 2.3. Preparation of the Green Tea Extract

GT (Alwazah Swan Brand, 100% pure Nett 225 g) aqueous extracts prepared through the daily (home) technique using GT in the biology department, College of Science, Suez Canal University. The absorption coefficient (which is 2) of GT leaves was taken into account during the preparation of the aqueous extract, which was then diluted to a ratio of 1 : 10. GT leaves weighing 5 g were ground into pieces smaller than 1mm in diameter before being combined with 60 mL of boiling distilled water, allowed to cool, and then filtered through a fine muslin cloth under pressure before being dried in a water bath at 40°C until a paste was obtained [17]. This paste could then be dissolved in distal water and used at a dosage of 40 mg/kg/day [19].

### 2.4. Preparation of Pomegranate Peel Extract

By hand, PPE was made by taking off the peel and cutting it into small pieces (2 cm × 2 cm). The parts will be frozen and dried (Free Zone Freeze Dry Systems) for 5 days in the Biology Department of the College of Science at Suez Canal University. Powder was made from the freeze-dried samples by using a mincer (FOSS Cemotec minced, Germany), and the powder was stored at 40°C until usage. Dry powder was mixed with distilled water (50-g dry solids/100 mL) and stored at 4°C until utilized [20].

All groups of rats were abandoned 1 week after the experiment with no treatment. At the end of the procedure, cervical dislocation was used to euthanize all the rats used in the study. The submandibular salivary gland was extracted and fixed in 10% of formalin to be prepared for histological and immune-histochemical evaluation.

### 2.5. Histological Evaluation

Submandibular salivary gland is washed in phosphate-buffered saline (PBS), then the specimens were fixed as follows: 70%, 80%, and 96% of ethanol for 90 min of each, three times in 100% ethanol for 60 min each, twice in xylol for 90 min each, and finally in liquified paraffin for 2 hr at 60°C. Lastly, 4-*µ*m-thick tissue slices were cut utilizing a rotary microtome, fixed on glass slides, and stained eventually with hematoxylin and eosin (H&E) and prepared for light microscopy [21].

### 2.6. Immunohistochemical Evaluation

By using the standard labeled streptavidin–biotin approach to apply P53 antibody tissue sections were cut at 4 m and mounted on positively charged slides (Lab Vision Corporation, USA). Next, the sections were deparaffinized using xylene and rehydrate via three 5-min runs through progressively more dilute ethanol solutions (100%, 95%, and 70%). The slides were washed twice for 5 min each in distilled water and PBS.

Hydrogen peroxide (H_2_O_2_) in methanol at a concentration of 3% was used to inhibit endogenous peroxidase activity for 30 min at room temperature. PBS was then used to clean the slides. After that, 200 mL of 10 M citrate buffer (pH 6) was placed in plastic jars, and the slides were submerged in them (ready to use from DAKO). The jars were heated at 100°C for 15 min at full power, three times. The slides were localized at room temperature over time. Distilled water and then PBS were used to wash the slides for 5 min. Primary antibodies were diluted 1 : 100 and dropped onto tissue sections, which were then incubated at room temperature in a humid environment overnight. After drying the slides, they were washed with distilled water and PBS for 5 min. The secondary antibody was biotinylated and incubated for 30 min at room temperature. After that, the tissue pieces were given a 5-min wash in PBS. After 30 min at room temperature with peroxidase-labeled streptavidin added, the cells were rinsed in PBS. After being treated with 3,3′-diaminobenzidine (DAB) for 2–4 min to induce staining, the tissue sections were submerged in distilled water. Before being mounted with DPX and coated with plastic for examination, the slides were given 2–3 min baths in 95% alcohol, followed by 2–3 min baths in pure alcohol. To determine where the immunostaining was in the tissues, a light microscope was used to view the immune-stained slices. The density of brown staining in the nuclei and cytoplasm of acinar and ductal cells determined whether a segment was positive or negative. Light microscopy at a magnification of (×200) was used to calculate the area percentage of positive staining for P53 stains. Using a computerized system (Software Leica-Quin 500, Wetzler, Germany) comprising a color video camera, color monitor, and CBU of an IBM of personal computer coupled to the microscope, regions were counted to get the positive index which was used to measure the area percentage of positive cells. The image analyzer was initially automatically calibrated so that the program's pixel-based measurements could be directly translated into micrometers [22]. Immunohistochemistry (IHC) staining strength was ranked as follows: weak (0), moderate (1), strong (2), and extremely strong (3) [23]. There were two separate rounds of testing for the samples by Image J [24]. IHC profiler plugin was used to perform color deconvolution as the first step in the analysis. Once the DAB image was deconvoluted, the immunostaining intensity of acinar and ductal cells was evaluated.

Cell nuclei were chosen by hand, and the “mean gray value” parameter was used to quantify the staining intensity. For each sample, the mean staining intensity was calculated across all cells in five randomly chosen fields of view. In Image J, pixel intensity values range from 0 to 255, with 0 denoting the darkest shade of a color and 255 the lightest. According to the thresholds established by the developers of the IHC profiler plugin [24], the staining intensities of the samples were categorized as follows: strong (3) for measured intensities ranking from 0 to 60, moderate (2) for intensities ranking from 61 to 120, weak (1) for intensities ranking from 121 to 180, and negative (0) for intensities higher than 181.

### 2.7. Statistical Analysis

The following statistical tests were used to gather, calculate, and tabulate the data. To make sure that the current samples were distributed normally, the Shapiro–Wilk test was used.

Mean standard deviation is the descriptive statistic that was used. Between-group comparisons were made using one-way analysis of altered (ANOVA). Pairwise comparisons be situated run using Tukey's post hoc testing statistical significances was assumed at the 0.05 level. All tests for statistical significance (*P*-value < 0.05) were conducted in SPSS 26.0 for Windows (Statistical Program for the Social Sciences; Armonk, NY: IBM Corp).

## 3. Results

### 3.1. Hematoxylin and Eosin Findings

#### 3.1.1. Control Groups

**Figure 2:**
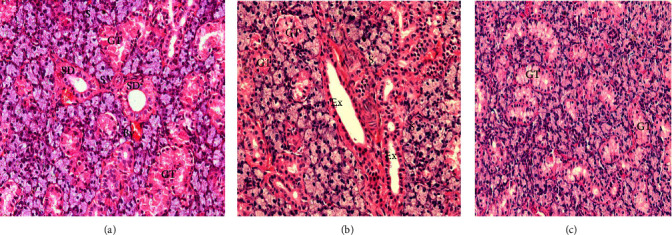
A photomicrograph of submandibular salivary gland of (a) vehicle control group, (b) GT extract control group, and (c) PPE control group. S, serous acinar cells; GT, granular convoluted tubules; Ex, execratory ducts; SD, striated ducts; and Bv, blood vessels (mag. ×400).

Most mammals do not have mucous or serous demilunes, and the submandibular glands of control subjects were only made up of well-arranged serous acini lined by pyramidal shaped cells with noticeable pale basophilic granular cytoplasm and round nuclei at the bottom. Intercalated ducts with granular convoluted tubules and striated ducts together with excretory ducts form up the duct system. Images of the submandibular glands from the *vehicle control*, *PPE control*, *and GT extract control groups* of rats showed normal structural architectures as control group with no discernible variations under the microscope (Figure 2).

#### 3.1.2. The MTX Treated Group

**Figure 3:**
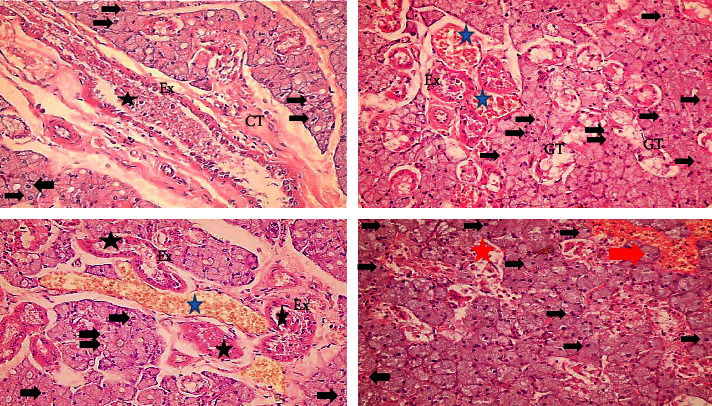
A photomicrograph of MTX group showing cytoplasmic vacuolization of gland elements. Oncocytosis of some acinar cells (black arrows); dilation of execratory ducts cells with stagnant secretion (Ex), granules and rounded cells in the lumen of ducts (black stars) fibrosis of connective tissue septa (CT); congestion and dilation of blood vessels (blue stars); areas of hemorrhage (red arrow); and area of complete loss of gland elements (red star) (mag. ×400).

Exhibited disorganization and loss of normal architecture of the submandibular salivary gland tissues. The serous acini and granular convoluted tubules had irregular outlines, and some acinar cells lost their cell boundaries. A significant haemorrhage was seen in the spaces between the acinar cells, and the cytoplasm of the acinar cells was slightly stained. There were little pyknotic nuclei, and the nuclei were moved to the periphery by vacuoles of varying sizes. Occasionally, some acinar cells showed nuclei with oncocytosis. Focal areas of acini destruction with areas of complete loss of acinar, granular convoluted tubules, and ductal cells. Ducts showed dilation and stagnated secretion, granulocytes and round cells can be observed in the ductal lumen. Connective tissue septa demonstrated thickening, fibrosis, and congested blood vessels (Figure 3).

#### 3.1.3. GT Extract + MTX Treated Group

Submandibular salivary gland showed almost normal acinar cells with basophilic cytoplasm, well-defined cell boundaries, and few cytoplasmic vacuolization. Ducts appeared with normal cell linings unless some ducts revealed stagnation in their lumen.

**Figure 4:**
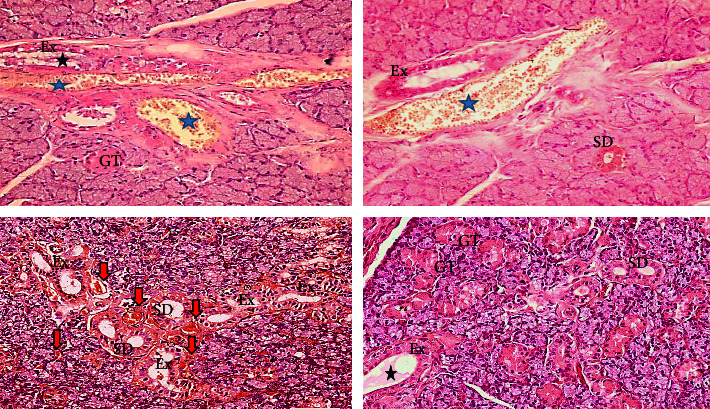
A photomicrograph of GT extract + MTX group showing normal cell lining of execratory ducts (Ex) and striated duct (SD). Dilated blood vessels engorged with RBCs (blue star); normal granular convoluted tubules (GT); stagnant secretion of the lumen of some ducts (black star); and new blood vessels in-between acinar and ductal cells (red arrows) (mag. ×400).

Moreover, angiogenesis was recorded in some samples surrounding ducts. Granular convoluted tubules appeared normal with few cytoplasmic vacuolization. Connective tissue septa appeared normal; however, dilation of blood vessels engorged with RBCs was recorded (Figure 4).

#### 3.1.4. PPE + MTX Treated Group

**Figure 5:**
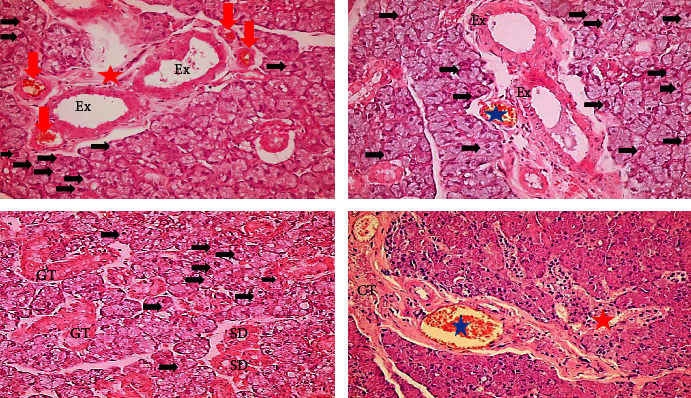
A photomicrograph of submandibular salivary gland of PPE + MTX group showing cytoplasmic vacuolization of gland elements. Oncocytosis of some acinar cells (black arrows); new blood vessels (red arrows); almost normal execratory (Ex) and striated ducts (SD) with potent lumen; dilated blood vessels engorged with RBC (blue star); fibrotic connective tissue septa (CT); and areas of complete loss gland elements with cellular infiltration (red star) (mag. ×400).

Revealed degeneration in the gland elements with disorganized, widely separated acini, serous acinar, and granular convoluted tubules that showed cytoplasmic vacuolization of variable size. Moreover, oncocytosis of some acinar cells were recorded. Areas of complete gland architecture loss with remnant of gland elements and hemorrhage. Both striated and excretory ducts appeared almost with normal cell lining and lumen unless there was some cytoplasmic vacuolization. Connective tissue septa appeared thickened and fibrotic, with congested blood vessels and low-grade inflammatory infiltration. Angiogenesis around ducts was recorded in some samples (Figure 5).

#### 3.1.5. Immunohistochemical Findings

**Figure 6:**
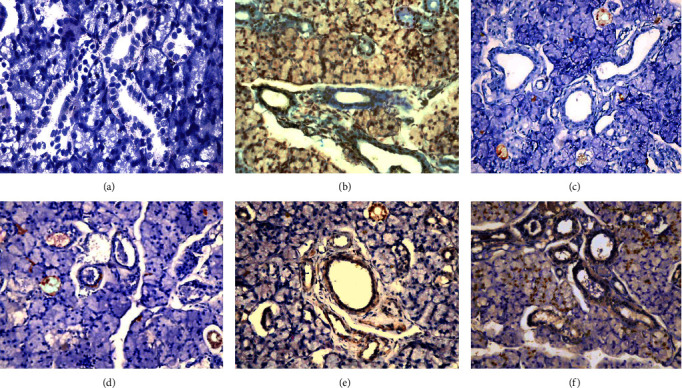
A photomicrograph of submandibular salivary gland of (a) vehicle control group with negative P53 immunoexpression, (b) MTX group nuclear and cytoplasmic immunoexpression of P53 antibodies, (c) both GT extract control group, (d) PPE control group with a localized nuclear P53 immunoexpression, (e) both GT extract + MTX group, and (f) PPE + MTX group with mild nuclear immunoexpression of P53 antibodies (mag. ×400).

Vehicle control showed negative expression of P53 antibodies both the GT and PPE control groups showed localized nuclear immunoexpression of P53 antibodies. MTX group demonstrated moderate nuclear and cytoplasmic immunoexpression to P53 antibodies; however, GT extract + MTX and PPE + MTX showed mild nuclear immunoexpression to P53 antibodies (Figure 6).

### 3.2. Statistical Analysis

The results in Table 1 showed that there are clearly significant differences between groups for P53 immunolocalization using one-way ANOVA (*F* = 687.012, *P*  < 0.001) at a significant level *P*  < 0.05. The pairwise comparison showed a significant difference between all groups except for vehicle control group with GT extract control group (*P*=0.986), vehicle control with PPE control group (*P*-value=0.999) and GT extract control group with PPE control group (*P*=0.995).

**Figure 7:**
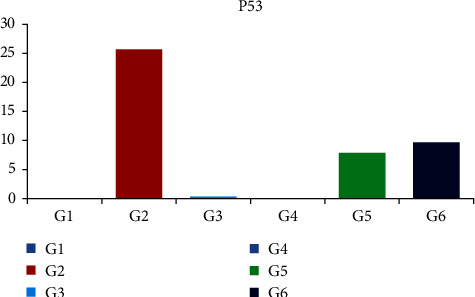
A chart represents the difference in mean of P53 optical density between different groups.

The MTX group recorded the highest mean value (25.69 ± 2.07) followed by PPE + MTX group (9.651 ± 1.09) and GT extract + MTX group (7.883 ± 1.17), while vehicle control group, GT extract control group, and PPE control group were the lowest mean value (Tables 1 and 2, Figure 7).

## 4. Discussion

Salivary glands are used as a research tool to investigate basic pharmacological questions. There is a strong correlation between the health of the mouth and the health of rest of the body because of the saliva produced by your salivary glands [25]. Exposure to chemotherapeutic doses of MTX, a folic acid antagonist [23], causes the salivary gland tissue to lose its function on a regular basis, with a significant drop in saliva output.

Since it is the second largest salivary gland, the submandibular gland was selected. It is responsible for making over 60% of the saliva in the body. Mucositis, extensive dental cavities, pharyngeal infections, and problems with swallowing, speech, and taste are all symptoms of secretory hypofunction, which could affect individuals undergoing cancer therapy or radiation therapy [26].

The mechanism of action of MTX, a folic acid antagonist, is to reduce the activity of many folate-dependent enzymes, thereby inhibiting DNA synthesis, repair, and cell proliferation. Cancer patients of all ages benefit from this drug's effectiveness as a chemotherapeutic agent. Extramedullary infiltration and systemic consolidation can be effectively treated in children with acute lymphoblastic leukemia by using high-dose MTX. However, HD-MTX limits its use due to substantial toxicity that occurs in most individuals treated with it [27]. The root of the MTX toxicity causes increased systemic oxidative stress; however, GT extract [12] and PPE [18] both include antioxidants and have anti-inflammatory characteristics, which may serve to shield the body from oxidative stress. Numerous interested research is raising in using natural antioxidant present in many food stuffs for preventing diseases caused by oxidative stress. Natural antioxidants are well-known for their safety and low costs [28]. Many studies have shown that the polyphenolic fractions isolated from GT extract inhibit oxidant stress and possess anti-inflammatory activity [29–31].

Scientists have discovered that PEE has the strong antioxidant activity [32, 33]. Punicalagin (PC) aellagic acid (EA) have been found to be the primary constituents of PPE antioxidant capabilities [34, 35]. Accordingly, purpose of the research was to compare the protective effects of GT and PPEs on submandibular salivary gland injury induced by MTX.

The current study results suggest that a single excessive dose of injected MTX can hurt the histological of the submandibular glands in rats by stopping or messing up protein synthesis by depleting foliate cofactors. This can lead to the formation of cytolysosomes, which is a sign of apoptosis [36]. During MTX treatment, damage to the submandibular salivary gland (vacuolization of acinar and ductal cells, apoptosis of acinar cells with pyknosis of the nuclei, and loss of some acinar cells) may be caused by oxidative stress from free radicals. In the process of MTX's intracellular metabolism, it creates free radicals that react with the cell membrane. This causes the membrane to break down, which leads to the release of important scavenger enzymes (based on glutathione) into the serum. This indicates a negative effect of MTX on cellular integrity [37] and alters the normal blood concentration of this antioxidant enzyme.

Necrosis is always thought to follow the commencement of either apoptotic cell death or oncocytic cell death, as the word “oncosis” has been introduced by Majno and Joris [38] to the opposing pattern of cell death with cell swelling. The prior research explains why localized acinar cell oncocytic alterations were observed in the current study.

There was also significant bleeding between the acinar cells. The granular degradation, inflammatory cell infiltration, and vascular congestion of other acini were also rather noticeable. Hsu et al. [39] corroborated these findings by explaining them because of the oxidative stress caused by MTX. Its intracellular metabolism has been shown to deplete glutathione-based antioxidants and generate oxygen free radicals. Lipid peroxidation and subsequent lysis of organelles and plasma membranes result from this oxidant/antioxidant imbalance [40]. It is possible that the goal of these inflammatory responses is to increase blood flow to the damaged areas of the body.

Histological examination revealed dilatation of the duct system in the group that had been given MTX, which was in line with Omar et al.'s [41] study that reported, MTX therapy causes an inflammatory reaction that increases transendothelial permeability, and such dilatation and congestion may be linked to MTX's unfavorable effect on myoepithelial cells embracing the ducts. Moheb et al. [42] demonstrated that expansion stops secretion, which leads to problems with salivary function, poor saliva ejection, and xerostomia (dry mouth). Acute inflammation of the salivary glands can lead to abscess formation, as evidenced by the presence of granulocytes and round cells in the ductal lumen [43]. According to Braicu et al.'s [8] oxidative stress can cause the release of inflammatory cytokines, the differentiation of cells into myofibroblasts, and the deposition of extracellular matrix components, all of which may explain why fibrosis was more pronounced after MTX administration in connective tissue septa. Nevertheless, Al-Refai et al. [12] mentioned how the administration of MTX causes an increase in collagen, which ultimately result in the death of acinar and ductal cells.

Areas of cellular infiltration into glandular connective tissue were seen in the MTX group. The presence of mononuclear infiltrations in the parotid gland following MTX therapy was consistent with the findings of Omar et al. [41]. Because ROS damage connective tissue as well as DNA and the cell membrane, it is possible that oxidative stress is responsible for the cellular infiltrations observed in the present study, which in turn stimulates macrophages, neutrophil infiltration, and pro-inflammatory cytokine release. Cure et al. [44] reported that MTX treatments resulted in enhanced subepithelial vascularity. Similarly, Zahawi [45] reported that in the submandibular salivary gland of rabbits, after chemotherapy, marked dilatation, and congestion of the blood vessels appeared in the MTX group. According to Omar et al. [41], this dilatation and congestion may be attributable to the inflammatory reaction associated with MTX treatment, which increases transendothelial permeability.

MTX, a chemotherapeutic medication, was found to have significant cytotoxic effects on the salivary glands in the present investigation, correlating with the findings of a previous study by Fawzy et al. [46].

In the present study, rats given MTX after GT extract experienced fewer changes in them salivary glands than those who received MTX alone. This could be because the flavonoids and polyphenols in GT can stabilize and keep the integrity of cell membranes. This helps cells grow back and makes it easier for proteins to be made, which repairs damaged tissues [47]. These results are in agreement with Al-Refai et al.'s [12] study that concluded, GT aqueous extract protected the submandibular salivary glands of rats against MTX-induced cytotoxicity, suggesting that it may be useful as a natural product to protect the salivary glands of people undergoing cancer therapy.

Moreover, Korany and Ezzat [48] investigated the preventive effects of GT against black seed on the rat parotid gland following a fenitrothion (pesticide) (a broad-spectrum insecticide) injection, GT had a calming effect on the parotid gland, which showed minimal cytoplasmic vacuolization and a generally normal arrangement of structure after tea therapy. The protective effect was attributed to its antioxidant characteristics, capacity to scavenge ROS, anti-inflammatory effects, and resistance to the drug's cytotoxic effects [48].

GT is a natural antioxidant, vitamin E, which serves to guard against the damaging effects of free radicals and may even boost the antioxidant potential of the tea [49]. Vitamins C and E work together to defend the cell membrane and stop peroxidation, which has an effect on the cell [50]. In addition, Khurana et al. [51] demonstrated that vitamin E has also been demonstrated to possessed antibacterial, antiviral, and antifungal properties. Thus, GT extract could help to minimize the severity of salivary gland damage due to MTX treatment.

On the other hand, PPE + MTX enhances the striated and excretory ducts' histological appearance that may be due to decreasing cytoplasmic vacuolization, but the combination of MTX and PPE did not resolve the structural damage in salivary gland as there were areas of complete loss of salivary gland elements, oncosytosis in acinar cells, and areas of blood vessels rupture and hemorrhage that consequently affect gland function. The present study findings were at odds with those of Shadab et al. [52] who found that an aqueous suspension of peel powder exhibited a potent antioxidant effect. Additionally, Sorrenti et al. [53] demonstrated that the ellagitannins and anthocyanins found in pomegranates contribute to their therapeutic benefits, as they offer protection against a wide range of disorders, including inflammatory ones. Pomegranate waste (peel and seeds) extracts, which are derived from industrial processing waste products, have been found by numerous researchers to exhibit strong antioxidant and free radical scavenger properties. Polyphenolic compounds with a content of punicalagins, gallic acid, and ellagic acid derivatives were also found to have notable antibacterial, antiviral, hypolipidemic, and anti-inflammatory bioactivities [53].

Although different studies on PPE applied on different tissues and diseases proved its protective effect, it was not promising in the present study that could be short applied duration or may not effective dose concentration comparing to the cytotoxic damaged occurred in the gland by MTX. Thus, it is recommended future research applying different doses concentration and different time intervals to reach the effective promising dose against the MTX cytotoxicity.

Nuclear transcription factor P53 regulates apoptosis, the cell cycle, and DNA repair, among many other biological processes. In addition to these classical roles, P53 has been shown to regulate intracellular redox homeostasis via transcriptional and nontranscriptional pathways, and this involvement appears to be increasingly essential. Common reactions to chemical exposure include the development of oxidative stress and activation of P53, both of which have been hypothesized to play crucial roles in chemical-induced toxicity. According to cellular responses to low or high levels of oxidative stress, activation of P53 can either exhibit pro-oxidant or antioxidant activity [54]. P53 displays antioxidant activities and increases cellular survival in response to moderate levels of oxidative-stress challenges; in reaction to increased levels of oxidative stress, P53 displays pro-oxidative actions to trigger apoptosis. Genomic stability can be preserved thanks to P53's dual ability to protect cells from oxidative damage. P53 performs these roles via modifying or directly regulating genes implicated in oxidative-stress responses via the P53 transcription factor [55].

In response to certain stress, P53 builds up in the nucleus and is moved to subnuclear domains (PML bodies) where it goes through more changes that turn it on. Stress also causes cytoplasmic P53 to move to the mitochondria, where it interacts with both pro- and antiapoptotic Bcl2 family members. This causes the mitochondria to release substances that promote apoptosis [56].

The levels of S-adenosyl methionine (SAM) and antioxidant enzymes like catalase, glutathione peroxidase (GPx), and superoxide dismutase in the cerebral fluid of MTX-treated individuals have been demonstrated to decrease. An increase in ROS may be the result of a SAM shortage that MTX causes ROS. Direct toxicity from increased ROS generation contributes to MTX's effects. Oxidative stress may occur due to imbalance between ROS creation and antioxidant defenses, and it can result in a number of clinical states [57].

Accordingly, P53 shows pro-oxidative activities by igniting prooxidative genes like proline oxidase and PIG3 [58], P53 triggers the expression of PUMA and BAX, which turns on apoptosis via the liberation of cytochrome c from mitochondria [59] and the current study suggested that the P53 immunoexpression in the MTX group could be due to the high level of oxidative stress confirmed by histological changes in salivary gland tissues. P53's prooxidative effects include suppressing antioxidant gene expression, which can enhance oxidative stress within cells and trigger apoptosis. The cytoplasmic expression in the MTX group, a group subjected to high cellular stress by MTX cytotoxicity, is an example of an apoptosis-inhibitory protein that is overexpressed during cellular stress [60].

Intranuclear, it turns off P53 tetramerization and increases its export, and intracytoplasmic, it neutralizes Bax to decrease cell death. Otherwise, the mild immunoexpression of P53 in the GT extract + MTX group which showed histological improvement could be a response to low levels of oxidative stress due to their antioxidants effect that stimulate P53 in antioxidant way. Otherwise, the PPE + MTX showed also mild P53 immunoexpression indicating stimulation of the antioxidant pathway but it was not enough to improve the degenerated tissue induced by MTX as revealed through the histological results.

Several P53 target genes, such as Sestrin, glutathione peroxidase (GPX), and aldehyde dehydrogenase (ALDH), help reduce oxidative stress [58]. These findings are in agreement with the studies of Zhao et al. [61] and Faraonio et al. [62] that suggested GT's antioxidants might raise levels of P53, a natural anticancer protein with the capacity to repair DNA damage or destroy cancerous cells. The previous study may also shed light on the localized expression of P53 in the GT extract and PPE control groups.

Studies by Yemelyanova et al. [63] supported the present study's hypothesis, reporting that factors contributing to weak P53 immunostaining include physiological accumulation of wild type P53, delayed P53 degradation under cellular stress, and regulatory defects between P53 and mouse double minute 2 homolog (MDM2). The source of weak IHC49 P53 positivity is unimportant [64].

Accordingly, the current study demonstrated the potent preventive effect of GT gland rather than PPE which showed nonpromising preventive effect against the MTX induced degeneration in submandibular salivary gland.

## 5. Conclusions

The antioxidant activity of GT extract in a dose (40 mg/kg/day orally) was more potent than that of PPE in blocking MTX-induced cytotoxicity in the submandibular salivary glands of rats. As a result, it can be administered to people undergoing cancer treatment as a safeguard for their salivary glands.

## Figures and Tables

**Table 1:** Comparison between different groups for P53 immunoexpression.

Groups	Mean	Standard deviation	*F*	*P*-value
Vehicle control group (G1)	0.0^d^	0.00	687.012	<0.0001 ^*∗∗*^
MTX group (G2)	25.69^a^	2.07		
GT extract control group (G3)	0.352^d^	0.32		
PPE control group (G4)	0.088^d^	0.11		
GT extract + MTX (G5)	7.883^c^	1.17		
PPE + MTX (G6)	9.651^b^	1.09		

Different letters (a, b, c, and d) and  ^*∗∗*^mean significant difference at *P* < 0.05.

**Table 2:** Pairwise comparison using Tukey's post hoc for P53 immunoexpression between different groups.

	Mean difference	*P*-values
Vehicle control group vs. MTX group	−25.69	<0.0001 ^*∗∗*^
Vehicle control group vs. GT extract control group	−0.35	0.98631
Vehicle control group vs. PPE control group	−0.09	0.99998
Vehicle control group vs. GT extract + MTX	−7.88	<0.0001 ^*∗∗*^
Vehicle control group vs. PPE + MTX	−9.65	<0.0001 ^*∗∗*^
MTX group vs. GT extract control group	25.34	<0.0001 ^*∗∗*^
MTX group vs. PPE control group	25.60	<0.0001 ^*∗∗*^
MTX group vs. GT extract group + MTX	17.81	<0.0001 ^*∗∗*^
MTX group vs. PPE + MTX	16.04	<0.0001 ^*∗∗*^
GT extract control group vs. PPE control group	0.26	0.99572
GT extract control group vs. GT extract + MTX group	−7.53	<0.0001 ^*∗∗*^
GT extract control group vs. PPE + MTX group	−9.30	<0.0001 ^*∗∗*^
PPE control group vs. GT extract + MTX group	−7.79	<0.0001 ^*∗∗*^
PPE control group vs. PPE + MTX	−9.56	<0.0001 ^*∗∗*^
GT extract + MTX group vs. PPE + MTX group	−1.77	0.02350 ^*∗∗*^

^*∗∗*^Means significant difference at *P* < 0.05 using Tukey's post hoc.

## Data Availability

Data are available upon request from corresponding author.
